# *Lactobacillus paracasei* DTA81, a cholesterol‐lowering strain having immunomodulatory activity, reveals gut microbiota regulation capability in BALB/c mice receiving high‐fat diet

**DOI:** 10.1111/jam.15058

**Published:** 2021-05-06

**Authors:** A. Tarrah, B.C. dos Santos Cruz, R. Sousa Dias, V. da Silva Duarte, S. Pakroo, L. Licursi de Oliveira, M.C. Gouveia Peluzio, V. Corich, A. Giacomini, S. Oliveira de Paula

**Affiliations:** ^1^ Department of Agronomy Food Natural Resources Animals and Environment University of Padova Viale dell’Universitá Italy; ^2^ Department of Nutrition and Health Federal University of Viçosa Viçosa Minas Gerais Brazil; ^3^ Department of General Biology Federal University of Viçosa Viçosa Minas Gerais Brazil

**Keywords:** 16S rRNA, genome sequencing, gut microbiota, immunomodulatory effect, probiotic, total cholesterol‐lowering

## Abstract

**Aims:**

*In‐vitro*/*In‐vivo* evaluation of cholesterol‐lowering probiotic strain *Lactobacillus paracasei* DTA81 and the possible connection with the gut microbiota modulation.

**Methods and Results:**

In the present study, strain DTA81 has been evaluated for the possible influence on blood lipid and glucose concentrations, modulation of the immune system, gastrointestinal survivability and modulation of gut microbiota in BALB/c mice receiving a high‐fat diet. After 6 weeks of treatment, a significant reduction of total cholesterol and fasting blood sugar (FBS) among animals treated with *L. paracasei* DTA81 has been recorded. Comparison of colon tissue levels of different cytokines revealed a significant reduction of the inflammatory cytokine interleukin‐6. The comparison of gut microbiota using the 16S rRNA approach indicated that the treatment with *L. paracasei* DTA81 significantly increased the taxa *Bacteroidetes* and *Coprococcus*. Moreover, the genome of DTA81 was sequenced for the *in‐silico* assessment, and the analysis indicated the presence of cholesterol assimilation‐related genes as well as the absence of negative traits such as transmissible antibiotic resistance genes, plasmids and prophage regions.

**Conclusion:**

The outcome of this study revealed the *in‐vitro* and *in‐vivo* properties of *L. paracasei* DTA81 and the possible mechanism between consumption of this strain, the abundance of *Bacteriodetes/Coprococcus* taxa, immunomodulatory activity and the subsequent reduction of cholesterol/FBS in BALB/c mice.

**Significance and Impact of the Study:**

*Lactobacillus paracasei* DTA81 as a non‐pharmacological potential probiotic supplement can influence metabolic homeostasis in individuals, particularly those adopting high‐fat diets, and it can contribute to reduce coronary heart disease.

## Introduction

According to the last definition by the FAO/WHO (Food and Agriculture Organization/World Health Organization), probiotics are ‘live microorganisms which when administered in adequate amounts confer a health benefit on the host’ (Hill *et al*. 2014). Probiotic foods represent a relevant portion of functional foods available on the market worldwide, projected to reach a value of US $ 46·55 billion by 2020 (Singh *et al*. 2018). During the past years, the probiotic potential of many lactic acid bacteria has been studied since they are generally recognized as safe micro‐organisms and can, therefore, be safely used in food preparations (Tarrah *et al*. 2020a, 2020b). Probiotic consumption can benefit human health by modulating the immune system, affecting the gut microbial composition and by producing antimicrobial substances that can contribute to the reduction of deleterious bacteria and promote stability of beneficial microbes (Magnusson and Schnürer 2001; Baker *et al*. 2009; Amund 2016). Many studies revealed the influence of gut microbiota on metabolic disorders and obesity in humans (Crovesy *et al*. 2020; Wang *et al*. 2020). A recent study indicated that energy homeostasis and metabolism of the human can be directly influenced by the gut microbiota (Cani *et al*. 2019). Indeed, new studies report that transferring the gut microbiota from obese to germ‐free mice can result in a higher weight increase in comparison to the transfer of gut microbiota from a lean mouse (Rosenbaum *et al*. 2015; Cani *et al*. 2019). Nowadays, hypercholesterolaemia is reported to be a common human disorder, which is mostly related to cardiovascular disease (CVD) and coronary heart disease (CHD) (Dunn‐Emke *et al*. 2001). Many *in‐vitro* and *in‐vivo* studies recently reported that probiotics such as some *Lactobacillus* and *Bifidobacterium* can have beneficial effects on serum lipid profiles (He *et al*. 2017; Mo *et al*. 2019). Probiotics can also reduce blood cholesterol in different ways, by utilizing prebiotics to produce short‐chain fatty acids (SCFAs) in the human gut that can further inhibit hepatic cholesterol synthesis and will result in a reduction of blood lipids or by assimilating cholesterol directly and reduce its presence in the human gut (Pereira and Gibson 2002). Therefore, several probiotic bacteria have been proposed and used as food supplements to reduce the rate of hypercholesterolaemia in humans (Marchesi *et al*. 2016). On the other side, it has been proven that probiotic strains can modulate the human immune system in different ways. The expression of cytokines in the human body has been the most frequent approach to describe the immunomodulatory effect of probiotics. In Wang *et al*.'s (2015) study, treated people with *Bifidobacterium* *bifidum*, *Bifidobacterium catenulatum*, *Bifidobacterium longum* and *Lactobacillus* *plantarum* showed a decrease in serum levels of pro‐inflammatory cytokines TNF‐α, IL‐5 and IL‐6, while levels of serum of IL‐10 significantly increased. In a previous *in‐vitro* study (Tarrah *et al*. 2019), we have characterized the probiotic potential of strain *Lactobacillus paracasei* DTA81 which was found to possess interesting properties. In particular, DTA81 revealed a strong adherence ability to human cell lines. Therefore, this strain was used in this study to be further investigated regarding its immunomodulatory effects, metabolic alteration and possible gut microbiota modulation effect, using *in‐vitro* and *in‐vivo* approaches.

## Materials and methods

### Bacterial strain and growth conditions

*Lactobacillus paracasei* DTA81 (Guerra *et al*. 2018) was routinely grown using MRS medium (Sigma, MO) at 37°C for 24 h. For *in‐vivo* assays, overnight cultures were centrifuged at 5000 ***g*** for 5 min, washed two times with sterile PBS (NaCl 8·0 g l^−1^, KCl 0·2 g l^−1^, Na_2_HPO_4_ 1·44 g l^−1^, KH_2_PO_4_ 0·24 g l^−1^, pH 7·4) and resuspended in skim milk (10%) to a final concentration of about 10^10^ CFU per ml.

### Measurement of cholesterol assimilation by *L. paracasei* DTA81

Initially, 1% overnight culture was incubated in MRS broth (Sigma) containing 0·30% ox gall bile salt (Sigma) and 100 *µ*g ml^−1^ filter‐sterilized cholesterol (Cholesterol–methyl‐β‐cyclodextrin, Sigma) for 24 h at 37°C. Tubes were then centrifuged at 5500 ***g*** for 15 min at 4°C, and 1 ml of supernatant was collected for measurement of residual cholesterol using a colorimetric method (Miremadi *et al*. 2014). Cholesterol concentration was measured using a standard curve from 0 to 100 *µ*g ml^−1^. The experiment was repeated two times with three replicates each. The ability of *L. paracasei* DTA81 to assimilate cholesterol was calculated as a percentage of cholesterol removal after 24 h.

### Animals

In all, 24 male BALB/c mice (4‐week old) were obtained from the Animal House at the Biological Sciences Center of the Universidade Federal de Viçosa. All mice were housed (four animals per cage) in a controlled environment: temperature 22°C, humidity 55 ± 5%, 12 h light/dark cycle and received food (Nuvilab, São Paulo, Brazil) and sterilized tap water *ad libitum* except at sampling time when the access to food was restricted. Mice body weight and food consumption were recorded weekly and daily respectively. The animal study design was approved by the animal ethics committee at the Universidade Federal de Viçosa (CEUA/UFV, protocol nº 15/2020) and it was in accordance with the National Research Council guide for the care and use of laboratory animals (Clark *et al*. 1997).

### Diets and experimental design

All animals were fed for a week (week 0) with a conventional diet (CD). Then they were randomly divided considering the body weight and GTT (glucose tolerance test) into three experimental groups (8 animals per group). The first one received the CD (CD group), the second one was fed with a high‐fat diet (HFD group) and the third one received HFD + *L. paracasei* DTA81 (DTA81 group). The composition of CD and HFD diets is reported in Table 1 (Reeves *et al*. 1993; Zhao *et al*. 2019).

**Table 1:** Composition of basal diets for conventional and high‐fat diet (g/100 g)

Ingredient	Conventional diet	High‐fat diet
Corn starch	46.56	–
Fat (lard)	–	31.7
Casein	14	25.8
Dextrinized starch	15.5	16.2
Sucrose	10	8.9
Soybean oil	4	3.2
Microfine cellulose	5	6.5
Mineral mix	3.5	1.3
Vitamin mix	1	1.3
L‐cystine	0.18	0.39
Choline bitartrate	0.25	0.3
Potassium citrate	–	2.1
Calcium phosphate	–	1.7
Calcium carbonate	–	0.7
Energy density (kcal g^−1^)	3.76	5.17

The potential probiotic strain was administered daily for 6 weeks (from weeks 1 to 6) via a unique oral administration by gavage of 100 *μ*l, equivalent to approx. 10^9^ CFU dispersed in 10% skim milk. During the same period, the remaining groups received the same amount of skim milk without cells. Then, the animals were anesthetized using ketamine (Imalgène, 200 mg kg^−1^, Sigma) and Rompun (Xylasine, 20 mg kg^−1^, Sigma) diluted in NaCl 0·9%, the mice were sacrificed by cervical dislocation, and the blood samples were collected from the retro‐orbital sinus for the biochemical analysis.

### Oral glucose tolerance test

The oral glucose tolerance test (OGTT) was performed at the end of week 0 and 6 (end of the experiment), according to Ferrere *et al*. (2016), with some modifications. A solution of 0·2% d‐glucose was given to each animal via gavage after overnight (12 h) fasting conditions, then blood was collected from the tail after 0, 30, 60 and 120 min. Glucose concentration in the blood serum was measured by putting a blood drop on a Comfort Curve Strips (F. Hoffmann–La Roche, Basel, Switzerland) that was then inserted into an ACCU‐CHEK Advantage Glucometer (Roche, Basel, Switzerland) and the OGT was determined according to Cardoso *et al*. (2011).

### Viable bacteria enumeration after transit through the GIT

The survival of *L. paracasei* DTA81 after transit through the GIT was evaluated at the end of week 3 and at the end of the study (end of week 6). Three mice from each group (from different cages) were randomly selected and their faeces were collected, weighed resuspended in 10 ml of sterilized PBS and serially diluted using the same solution. Then they were plated on MRS medium supplemented with kanamycin (64 *μ*g ml^−1^; Sigma) and incubated at 37°C for 48 h. Resistance of *L. paracasei* DTA81 to kanamycin had been determined in a previous study (Tarrah *et al*. 2019). After incubation, colony forming units were counted and reported per gram of wet faeces. Besides, five colonies were also randomly taken from plates and investigated by Gram staining, catalase and oxidase tests. The same mice were used for this evaluation at week 3 and at week 6.

### Determination of the lipid profile and transaminases

Blood samples collected from sacrificed animals were centrifuged at 700 ***g*** for 10 min to obtain the serums and immediately examined for total cholesterol, high‐density lipoprotein (HDL), triglyceride, glutamate‐oxaloacetate transaminase (GOT), and glutamate‐pyruvate transaminase (GPT) using Bioclin kits (Diagnostics, Belo Horizonte, Brazil) and an auto‐analyzer equipment (Analyzer BS‐200; Mindray, Shenzhen, China). Low‐density lipoprotein (LDL) was calculated according to the method of Friedwald et al. (Friedwald 1972).

### Immunomodulatory effects on colon tissue

The commercial BD CBA Human Th1/Th2/Th17 Cytokine Kit II (BD Biosciences, San Jose, CA) and a BD FACSVers flow cytometer were used to quantitatively measure interleukin‐2 (IL‐2), interleukin‐4 (IL‐4), interleukin‐6 (IL‐6), interleukin‐10 (IL‐10), interleukin‐17 (IL‐17), tumour necrosis factor (TNF) and interferon‐γ (IFN‐γ) protein levels in mice colon samples. To perform local inflammatory cytokine analysis, colon tissue (100–200 mg) was ground using a tissue homogenizer (IKA WORKS GMBH & CO, Staufen, Germany, model T10 basic), washed in cold phosphate‐buffered saline (pH 7·0) and centrifuged (10 000 ***g***, for 10 min at 4°C). Finally, the supernatant was collected and stored at −80°C for further analysis. The samples were prepared according to the manufacturer’s instruction before being analysed by flow cytometry.

### 16S rRNA gene amplicon target sequencing

Four mice from each group (from different cages) were randomly chosen at the beginning and at the end of the experiment and their faeces were collected in a separated clean cage for each mouse and the samples were immediately stored at −80°C for further analysis. Later, 0·25 g of collected faeces was weighed and total DNA was extracted using the DNeasy PowerSoil Microbial Kit (Qiagen, Valencia, CA) according to the manufacturer's instructions. The quality and quantity of the extracted DNA were assessed by the Spark 10 M spectrophotometer (Tecan Trading AG, Männedorf, Switzerland). The V3–V5 regions of the 16S rRNA gene were PCR amplified and sequenced using an Illumina MiSeq desktop sequencer (Eurofins Genomics Germany GmbH, Ebersberg, Germany) producing 300 bp paired‐end (PE) reads.

### Genome sequencing and genomic analysis of *L. paracasei* DTA81

*Lactobacillus paracasei* DTA81 was grown overnight in MRS broth at 37°C for 24 h and genomic DNA was isolated using the DNeasy PowerSoil Microbial Kit (Qiagen, Valencia, CA) according to the manufacturer's instructions. The assessment of isolated DNA quality and quantity was done using Spark 10M spectrophotometer (Tecan Trading AG, Männedorf, Switzerland). *Lactobacillus paracasei* DTA81 genome was sequenced using the paired‐end sequencing technology with NextSeq500 Illumina sequencer at the Interdepartmental Center for Research on Innovative Biotechnology CRIBI (CRIBI, Padova, Italy).

### Bioinformatic analyses

The raw data of 16S rRNA gene sequencing were imported and analysed with the CLC Genomics Workbench software v.12.0.2 (Qiagen, Hilden, Germany) using the Microbial genomics module plugin as described by da Silva Duarte *et al*. (2020). In summary, quality filtering, operational taxonomic unit (OTU) clustering, taxonomical assignment (Greengenes v13_8 database), alpha‐ and beta‐diversity indices calculation were performed with default parameters. Raw reads were deposited in the Sequence Read Archive (SRA) database (http://www.ncbi.nlm.nih.gov/sra) under the BioProject ‘PRJNA638135’.

The Shannon and Chao 1 indices were compared among the experimental groups (CD, DTA81 and HFD) using ANOVA and Kruskal–Wallis tests, followed by Tukey's and Dunn’s *post hoc* tests, correspondingly. The Welch’s *t* test was chosen to identify significant differences inside the same group (intra‐group comparison) between the time‐points ‘t0’ and ‘t1’. Both statistical analyses were conducted using GraphPad Prism software (version 7, GraphPad Software, Inc., San Diego, CA). For beta‐diversity analysis, gut microbial dissimilarities among the groups were calculated by permutational multivariate analysis of variance (PERMANOVA) with 999 permutations using Unweighted and Weighted UniFrac diversity metrics. Principal coordinate analysis (PCoA) was chosen as the ordination method to explore and visualize the data.

After data inspection, data filtering (low count and low variance filters) and normalization (cumulative sum scaling), differential abundance analysis were carried with MicrobiomeAnalyst (Dhariwal *et al*. 2017) considering three taxonomic levels (phylum, family and genus) by applying the linear discriminant analysis (LDA) effect size (LEfSe) function setting up FDR‐adjusted p value (*q* value) cutoff of 0·05 and log LDA score of 2·0. Pearson correlation with a complete linkage method was chosen to cluster the samples based on taxon abundance in a heat map graph generated with Heatmapper (Babicki *et al*. 2016).

Regarding DTA81 genome analysis, *de novo* assembly of the raw reads was done using Velvet algorithm package, ver. 1.1.04 setting on parameters as (min read length: 15, min average quality of read: 20 and min adapter match: 15) (Larsen *et al*. 2012). Rapid Annotation using Subsystems Technology (RAST) was also used for gene prediction and annotation (Aziz *et al*. 2008).

The PATRIC 3.6.3 server (Wattam *et al*. 2017) was used to construct a graphical genome map/annotation after scaffolding the related contigs using the Medusa web server (Bosi *et al*. 2015) and *L. paracasei* ATCC 334 as the reference genome.

The presence of prophage regions was predicted using the PHASTER server (Arndt *et al*. 2016). The detection of plasmid and transmissible antibiotic resistance genes was assessed using PlasmidFinder 2.0 and ResFinder 3.2 servers, respectively (Zankari *et al*. 2012; Carattoli *et al*. 2014).

This Whole Genome Shotgun project has been deposited at DDBJ/ENA/GenBank under the accession JAAVWK000000000. The version described in this paper is version JAAVWK010000000.

### Statistical analyses

Data were analysed using one‐way analysis of variance (ANOVA). Tukey’s test was used as *post hoc* analysis by the GraphPad Prism software (version 7, GraphPad Software, Inc., San Diego, CA). In general, results were considered significantly different when *P* values were lower than 0·05. The number of asterisks is used to indicate the level of confidence of the statistical analyses results: *P < 0·05, **P < 0·01, ***P < 0·001.

## Results

### *In‐vitro* cholesterol assimilation

Cholesterol reduction by *L. paracasei* DTA81 grown in MRS supplemented with 0·3% ox bile was measured *in‐vitro*. DTA81 was able to reduce 16·16 ± 2·0% of the cholesterol presents in the medium after 24 h.

### Weight gain and oral glucose tolerance

**Figure 1:**
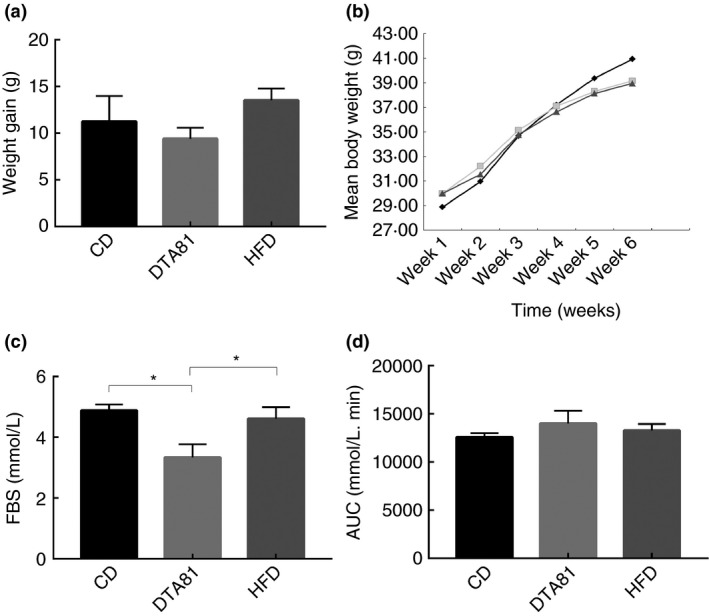
Effect of probiotic consumption on body weight, fasting blood sugar (FBS) and glucose tolerance test. (a) Mean body weight; (b) weight gain at the end of the experiment; (c) fasting blood sugar (FBS); (d) glucose tolerance test. Results are expressed as means ± SEM (*n* = 8). The number of asterisks is used to indicate the level of confidence of the statistical analyses results: *statistically significant *P* < 0·05, (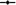) HFD; (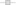) DTA81; (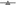) CD.

At the end of the experimental period (6 weeks), mice treated with DTA81 did not evidence a significant difference in weight gain (Fig. 1a); the weekly monitoring of animal weight (Fig. 1b) showed a ponderal increase of HFD during the last 2 weeks while DTA81 group had the same trend of CD. The influence of DTA81 supplementation on plasma glucose is indicated in Fig. 1c,d. Regarding the fasting blood sugar (FBS), a significant glucose reduction was recorded in the animals treated with *L. paracasei* DTA81 compared with the CD and HFD groups (*P* < 0·05) (Fig. 1c). However, after receiving the glucose, no significant difference was recorded regarding the glucose tolerance among the groups, neither at the beginning nor at the end of the experiments (Fig. 1d).

### Determination of the lipid profile and transaminases

**Figure 2:**
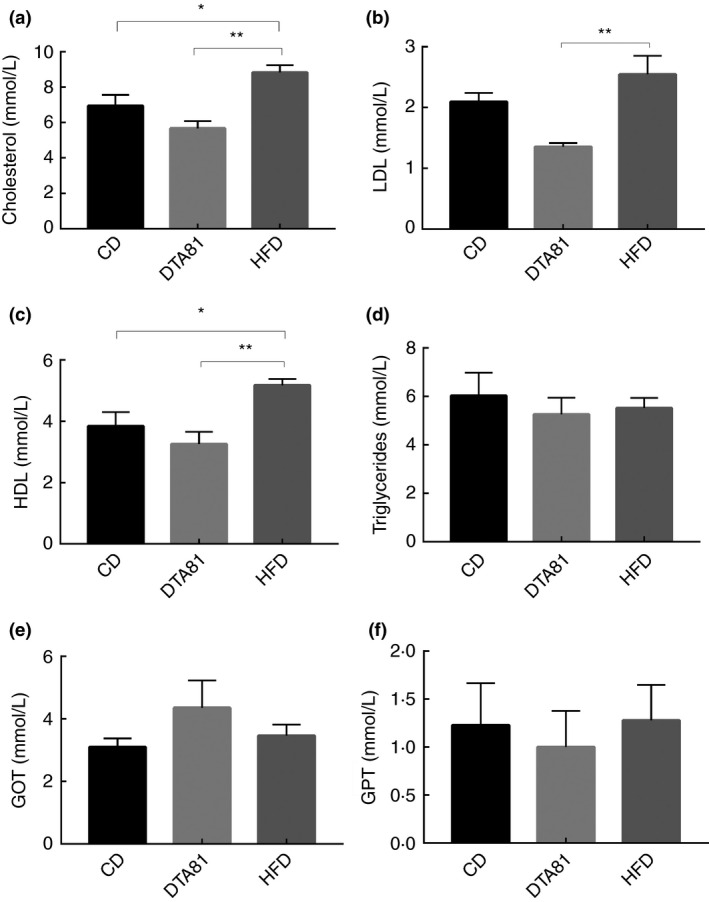
Effect of different treatments on blood parameters. (a) Total cholesterol; (b) high‐density lipoprotein (HDL); (c) low‐density lipoprotein (LDL); (d) triglyceride; (e) glutamate‐oxaloacetate transaminase (GOT); (f) glutamate‐pyruvate transaminase (GPT); Results are expressed as means ± SEM (*n* = 8). The number of asterisks is used to indicate the level of confidence of the statistical analyses results: *statistically significant *P* < 0·05, **statistically significant *P* < 0·01.

Results of mice blood biochemical analyses at the end of the study (week 6) are reported in Fig. 2. Total cholesterol (TC), HDL and LDL evidenced a significant (*P* < 0·01) reduction in the DTA81 group compared with the HFD group, whereas triglycerides, GOT and GPT did not show significant differences between any group. In addition, a significant difference (*P* < 0·05) was detected for TC and HDL between groups HFD and CD while no statistically significant difference was seen for LDL.

### Immunomodulatory activity in the colon tissue

**Figure 3:**
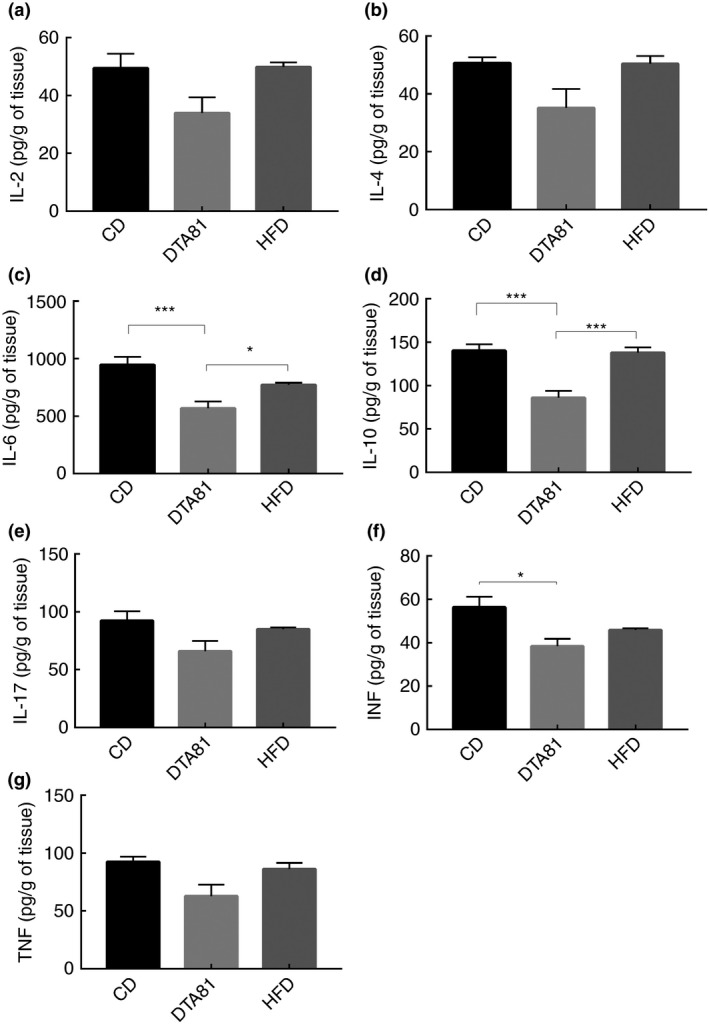
Effect of different treatments on local cytokines. (a) Interleukin‐2; (b) interleukin‐4; (c) interleukin‐6; (d) interleukin‐10; (e) interleukin‐17; (f) interferon gamma; (g) tumour necrosis factor. Results are expressed as means ± SEM (*n* = 8). The number of asterisks is used to indicate the level of confidence of the statistical analyses results: *statistically significant *P <* 0·05, ***statistically significant *P* < 0·001.

Comparison of colon tissue levels of Th1 (IL2, TNF and IFN‐γ), Th2 (IL4, IL6 and IL10) and Th17 (IL‐17) at the end of the experiment (week 6) is presented in Fig. 3. The outcome revealed a significant reduction of IL‐6 and IL‐10 in the group treated with *L. paracasei* DTA81 when compared with CD and HFD groups. However, the level of cytokines IL‐2, IL‐4, IL‐17, TNF and INF‐γ did not show any significant difference between DTA81 and HFD or CD groups, except for INF‐γ that showed a significant difference with the CD group.

### 16S rRNA sequencing analysis of the gut microbiota

A high‐throughput 16S rRNA sequencing was performed to assess possible changes in the gut microbiota composition caused by the administration of high‐fat diet or by *L. paracasei* DTA81 supplementation. A total of 536 330 reads from mice faecal microbiota were analysed with a mean of 22 621 (±8·191) sequences for each sample (8 samples for each experimental group; *n* = 24). Shannon index and phylogenetic diversity curves (Fig. S1) confirmed that the total number of reads obtained from 16S rRNA gene amplicon sequencing covered most of the microbial diversity and that the majority of bacterial phylotypes inside each group were sampled.

**Figure 4:**
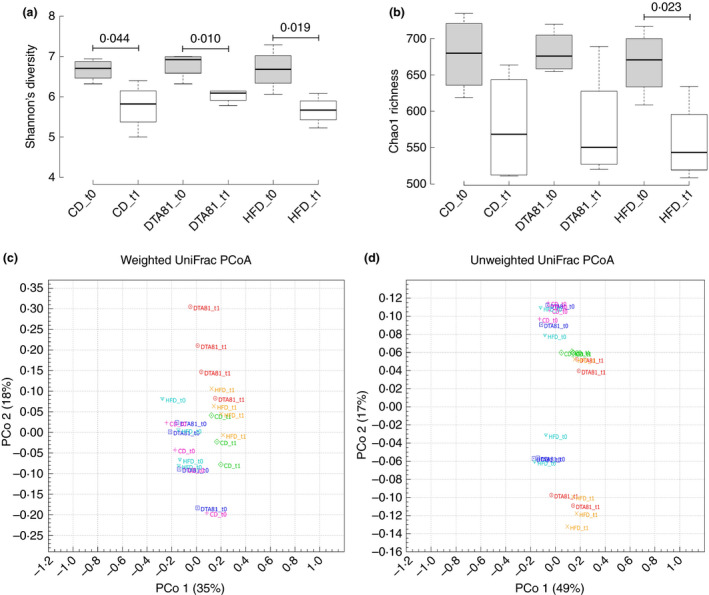
Alpha‐ and beta‐diversity analysis of faecal samples. Box and whisker plots comparing the alpha‐diversity indices Shannon diversity (a) and Chao1 richness (b) among the three groups (CD, DTA81 and HFD) before (t0) and after (t) their respective intervention. Horizontal bold lines show the median values. The bottom and top of the boxes show the 25th and the 75th percentiles respectively. The whiskers extend up to the most extreme points within 1·5 times the interquartile ranges (IQR). Level of significance: *P* ≤ 0·05. Principal coordinate analysis (PCoA) based on Weighted (c) and Unweighted (d) UniFrac distances for CD, DTA81 and HFD in two different time‐points (t0 and t1). PERMANOVA with 999 permutations was used to detect significant microbial community structure dissimilarity in the different experimental groups enrolled in this study. , (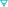) HFD_t0, (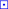) DTA81_t0, (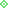) CD_t1, (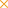) HFD t1, (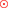) DTA81 t1, (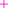) CD_t0.

At the end of the experimental period (t1), a significant reduction in faecal microbial diversity was observed inside all groups (Fig. 4a), as evidenced by Shannon's diversity index, compared to the same groups at the beginning of the experiment (week 0; t0). A reduced Chao1 value (richness diversity index) was also detected at the end of the experimental period in faecal samples of all animals regardless the experimental group. However, only animals of the HFD group displayed a significant reduction in diversity compared to the same group at the beginning of the trial (Fig. 4b).

The investigation of community structures of faecal samples of BALB/c mice was done using the phylogenetic distance‐based measurements weighted and unweighted UniFrac. As shown in Fig. 4c,d, scatter plot of the PCoA using both distance metrics revealed a significant microbial shift (PERMANOVA weighted UniFrac: *P* = 0·001, Pseudo‐*f* statistic = 4·87; PERMANOVA unweighted UniFrac: *P* = 0·001, Pseudo‐*f* statistic = 2·24) after the experimental period in all groups enrolled in this study, although a more pronounced difference was observed when weighted UniFrac distance metric was considered, which accounts for the relative abundance of OTUs. Supplementation of strain DTA81 significantly changed the intestinal microbial composition when compared to the groups receiving a CD (PERMANOVA weighted UniFrac: *P* = 0·029, Pseudo‐f statistic = 4·49; PERMANOVA unweighted UniFrac: *P* = 0·031, Pseudo‐*f* statistic = 1·50) and an HFD (PERMANOVA unweighted UniFrac: *P* = 0·027, Pseudo‐*f* statistic = 1·60). A significant difference in terms of beta‐diversity of the intestinal microbiota was also observed between CD and HFD groups (PERMANOVA weighted UniFrac: *P* = 0·023, Pseudo‐*f* statistic = 3·11; PERMANOVA unweighted UniFrac: *P* = 0·025, Pseudo‐*f* statistic = 1·60). In total, weighted and unweighted UniFrac components (PCoA 1 and PCoA 2) accounted, respectively, for 53 and 66% of the total variance.

**Figure 5:**
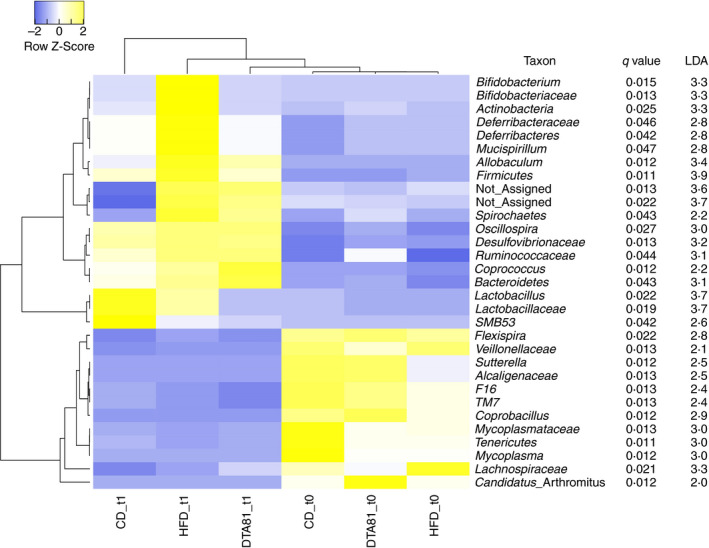
Heat map based on linear discriminant analysis effect size (LEfSe) at phylum, family and genus levels. An FDR adjusted *P* value (or *q* value) less than 5% and an LDA score greater than 2 were used to determine taxa that are significantly enriched among the groups CD, DTA81 and HFD before (t0) and after (t1) the experimental period. In the heat map, an enrichment trend is reported in yellow while depletion is represented in blue.

The relative distributions of bacteria at the phylum, family and genus level identified by 16S rRNA gene amplicon sequencing are reported in Fig. S2. Prevalent phyla considering all groups were *Firmicutes* (65%), *Proteobacteria* (16%) and *Bacteroidetes* (6%). At t1, the consumption of a high‐fat diet (HFD group) significantly increased *Firmicutes* (LDA = 3·9), *Actinobacteria* (LDA = 3·3), *Deferribacteres* (LDA = 2·8) and *Spirochaetes* (LDA = 2·2), whereas the supplementation of DTA81 significantly increased *Bacteroidetes* (LDA = 3·1) (Fig. 5). Considered important in the development of obesity, the *Firmicutes*/*Bacteroidetes* (F/B) ratio was calculated and results show that the potential probiotic intervention improved on average the proportion F/B (7·8 ± 4·65) when compared to the groups that received a CD (11·11 ± 3·95) or a high‐fat diet (18·71 ± 12·65).

At family level, Lachnospiraceae, Ruminococcaceae, Erysipelotrichaceae, Lactobacillaceae and Desulfovibrionaceae were the top five taxa (Fig. S2b). At the end of the experimental period, major changes were observed mainly in samples from the HFD group, where four families (Bifidobacteriaceae, LDA = 3·3; Desulfovibrionaceae, LDA = 3·2; Ruminococcaceae, LDA = 3·1; Deferribacteraceae, LDA = 2·8) resulted overrepresented in this group (Fig. 5). Only the family Lactobacillaceae increased in the CD group (LDA = 3·7), while in the DTA81 group there were no statistically significant biomarkers (LDA score <2 and/or *q* value >0·05) after the potential probiotic supplementation.

With regard to the genera observed in faecal samples across the groups, *Lactobacillus*, *Oscillospira*, *Allobaculum*, *Helicobacter* and *Ruminococcus* were identified as the top five taxa (Fig. S2c). Linear discriminant effect size (LEfSe) analysis revealed an enrichment of 13 biomarkers (Fig. 5), among which a higher relative abundance of the genera *Oscillospira* (LDA = 3·0) and *Coprococcus* (LDA = 2·2) were associated with *L. paracasei* DTA81 administration. In stool samples of mice of the CD group, an enrichment of the genera *Lactobacillus* (LDA = 3·7) and *SMB53* (LDA = 2·6) was observed at the end of the experimental period. Lastly, the HFD was associated with a higher proportion of *Allobaculum* (LDA = 3·4), *Bifidobacterium* (LDA = 3·3) and *Mucispirillum* (LDA = 2·8) in faeces samples.

### *L. paracasei* DTA81 cell enumeration after mice GI transit

Survival of *L. paracasei* DTA81 after passage through mice GIT was evaluated after 21 (week 3) and 42 (week 6) days. The strain was administered to the animals at the dose of approx. 10^9^ CFU per day and viable cells were then enumerated from collected faeces. After week 3, potential probiotic‐treated animals revealed 8·50 ± 0·16 log CFU per g of wet faeces while no colony was recovered from CDC and HFD groups. After week 6, no significant difference in the number of retrieved cells (8·67 ± 0·23 log CFU per g) was recorded.

### Genome sequencing and genomic analysis of *L. paracasei* DTA81

**Figure 6:**
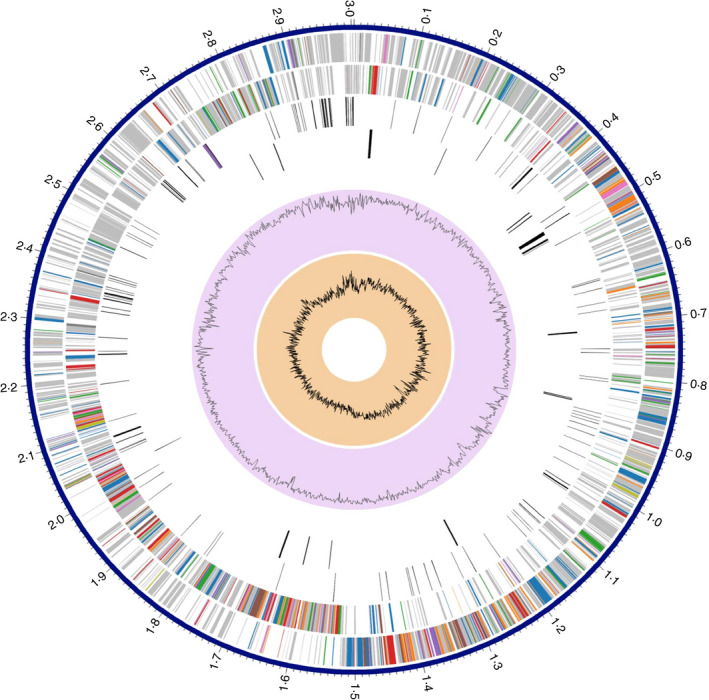
Circular graphical display of the distribution of the genome map and annotations. This includes, from outer to inner rings, CDS on the forward strand, CDS on the reverse strand, RNA genes, CDS with homology to known antimicrobial resistance genes, CDS with homology to known virulence factors, GC content and GC skew. The colours of the CDS on the forward and reverse strand indicate the subsystem that these genes belong to. (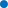) Metabolism, (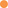) Protein processing, (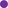) Stress response, Defence, Virulence, (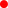) DNA processing, (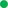) Energy, (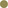) Cellular processing, (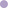) RNA processing, (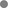) Membrane transport, (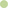) Cell envelope, (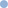) Regulation and cell signalling, (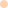) Miscellaneous.

Sequencing of *L. paracasei* DTA81 genome produced 1 443 422 reads with an average size of 150·5 bp. The assembled genome of *L. paracasei* DTA81 produced 39 scaffolds, giving a genome size of 3·00 Mb with a GC content of 46·1% (Table 2). RASTtk server predicted a total number of 3077 protein‐coding sequences (CDSs) classified into 236 different subsystems. The largest part of this subsystem is allocated to the carbohydrate metabolism (22·03%) followed by amino acids and derivatives (13·45%) and protein metabolism (11·86%). A total number of 59 structural RNAs including 3 complete rRNAs (5S, 16S and 23S) and 56 tRNAs were predicted, too. The average nucleotide identity (ANI) of 98·7% with the closest neighbour (*L. paracasei* ATCC 334) allowed a precise taxonomical placement of strain DTA81 inside the species *L*. *paracasei*. In Fig. 6, a circular graphical map of the distribution of the genome annotations of *L. paracasei* DTA81 is provided which indicates the location of CDS on the forward and reverse strands, RNA genes, antimicrobial resistance genes and virulence factors. A deep analysis within the genome of DTA81 has also revealed the presence of cholesterol assimilation‐related genes namely*, ccpA, fba*, *lbpg_rs09895*, *lbpg_rs11190* and *lbpg_rs10085* (Table 3) (Lee *et al*. 2010). Finally, the absence of mobile elements such as prophage regions, acquired antibiotic resistance genes and plasmid sequences on DTA81 genome was confirmed using PHASTER, PlasmidFinder and ResFinder servers respectively.

**Table 2:** Main characteristics of *L. paracasei* DTA81 genome

Feature	Value
Genome size	3 002 945
G+C content (%)	46.2
Contig N50	156 284
Contig L50	7
Number of scaffolds	39
Number of protein coding sequences (CDSs)	3077
Number of rRNAs	3
Number of tRNAs	56
Number of genes related to Virulence, disease, and defense	0

**Table 3:** Identification of cholesterol assimilation‐related genes in *Lactobacillus paracasei* DTA81

Gene	Gene description	Organism	Identity score (%)	Accession number
*ccpA*	Catabolite control protein A	*L. paracasei* AO356	100.00	CP025499.1
*fba*	Class II fructose‐1,6‐bisphosphate aldolase	*L. paracasei* N1115	100.00	CP007122.1
*lbpg_rs09895*	Glycogen phosphorylase	*L. paracasei* AO356	100.00	CP025499.1
*lbpg_rs11190*	FMN‐binding protein	*L. paracasei* W56	100.00	HE970764.1
*lbpg_rs10085*	MFS transporter	*L. paracasei* N1115	100.00	CP007122.1

## Discussion

It has been reported that the presence of probiotics inside the gut microbiota can be considered as a potential therapeutic strategy for some metabolic disorders such as hyperglycaemia, hypercholesterolemia and obesity (Janssen and Kersten 2015).

In this study, mice treated with DTA81 did not evidence a significant difference in weight gain. It should be noted that the use of a high‐fat diet does not always cause changes of total body weight, and metabolic changes related to obesity, such as hyperlipidaemia, hyperglycaemia, diabetes and low‐grade inflammation, should also be observed. In a recently published study, the GTT was recorded significantly higher in BALB/c mice that had received a high‐fat diet compared to BALC/c mice that received a control diet. This indicates that the high‐fat diet interfered with the glucose metabolism in these mice; besides, serum levels of total cholesterol, LDL‐c, triglycerides/HDL‐c ratio, liver and adipose tissue were higher in BALC/c mice that had received the high‐fat diet. However, there was no change in body weight (Li *et al*. 2020).

After 6 weeks of treatment, a significant fasting blood glucose reduction was recorded in the animals treated with *L. paracasei* DTA81 compared with the CD and HFD groups. The reduction of fasting blood glucose by DTA81 compared to the group receiving a CD is very interesting since the CD (control) contains higher carbohydrate concentrations than that of the high‐fat diet; therefore, our findings demonstrate the possible benefit of DTA81 in this condition too. The mechanism of glucose‐lowering by probiotics is not well understood, but probiotics can modulate the human immune system which can influence glucose metabolism. Moreover, probiotics can reduce the inflammatory cytokines and regulate the immune system (de LeBlanc and Perdigón 2010).

Laitinen *et al*. (2008) demonstrated that the immunomodulatory effect of probiotics can lead to glucose reduction. In our study, *L. paracasei* DTA81, with its fasting blood‐sugar‐lowering activity, induced significantly lower values for interleukin 6 and 10.

Probiotics can also lower blood cholesterol in different ways, indirectly by fermentation of prebiotics and consequent production of SCFA in the human gut that can further inhibit hepatic cholesterol synthesis and will result in a reduction of blood lipids (Ashaolu *et al*. 2020). Alternatively, probiotics can assimilate cholesterol directly, thus eliminating it from the human gut (Pereira and Gibson 2002; Pan *et al*. 2011; Öner *et al*. 2014). In their study, Shimizu *et al*. (2015) reported that consumption of probiotics by elderly and hypercholesterolaemic patients could be more effective than in youngsters and individuals with normal lipid levels. Besides, probiotics can reduce cholesterol levels by assimilating and entrapping this molecule into bacterial membranes (Castorena‐Alba *et al*. 2018; Bhat and Bajaj 2020). There are numerous species of *Lactobacillus* and *Bifidobacterium* that have shown cholesterol assimilation in *in‐vitro* experiments and it has been reported that this ability is strictly strain‐dependent (Costabile *et al*. 2017; Castorena‐Alba *et al*. 2018). Taking into account the published data in the literature, cholesterol assimilation by probiotic strains can range from 0·86% to more than 40%; however, we usually do not see the same reduction when we use probiotics in the *in‐vivo* conditions, which can be due to the effect of gastrointestinal conditions of the strains, microbial competition, colonization on the epithelial cells, etc. (Belviso *et al*. 2009; Tokatlı *et al*. 2015; Castorena‐Alba *et al*. 2018). The outcome of our study indicates that consumption of *L. paracasei* DTA81 can lead in mice to a statistically significant reduction of total cholesterol and LDL and HDL, which appears very interesting and useful in people who suffer from CVD and CHD. This ability by *L. paracasei* DTA81 could be connected to the presence of cholesterol assimilation‐related genes, coding membrane‐associated proteins that can adhere to the cholesterol molecule and further incorporate it inside the cell (Lee *et al*. 2010). Besides, in our previous study (Tarrah *et al*. 2019), DTA81 had revealed a great adhesion capability to human cell lines, which could play a significant role in its potential beneficial activity.

Gut microbiota dysbiosis can also promote the occurrence of metabolic syndromes in diverse ways, such as low‐grade inflammation, through increased production of bacterial lipopolysaccharide. In the present study, we detected an increase in *Bacteroidetes* and *Coprococcus* after 6 weeks of treatment with strain DTA81. The phylum *Bacteroidetes* belongs to Gram‐negative bacteria that normally colonize the human lower gastrointestinal tract during infancy, due to the abundance of non‐digestible oligosaccharides in mother’s milk which support their growth (Marcobal *et al*. 2011). Since colonization, they play an essential role by breaking down complex sugars and degrading proteins in the human gut, as well as by exerting an immunomodulatory effect (Rakoff‐Nahoum *et al*. 2004; Rajilić‐Stojanović and de Vos 2014). Another important function of this bacterial group in the human gut is related to the deconjugation of bile acids which is linked to cholesterol‐lowering activity (Narushima *et al*. 2006; Leitch *et al*. 2007). Also, it has been reported that, due to the broad metabolic potential of *Bacteroidetes*, their reduced abundance could be linked to obesity in humans (Ley 2010). On the other side, it was proven that the increase in the genus *Coprococcus,* an anaerobic genus which is normally present in the human faecal microbiota, can have an anti‐carcinogenic and anti‐inflammatory effect in the human gut, due to butyric acid production (Hamer *et al*. 2008; Ai *et al*. 2019).

As determinants and modulators of immune pathology, cytokines play a key regulatory role among the many components of the animal immune system (Lin and Karin 2007). A broad spectrum of cells such as fibroblasts, endothelial cells, neuronal cells, macrophages and mast cells can produce cytokines; however, production is mainly dependent from the differentiation state of T cells, which can be divided into three different types according to the pattern of cytokine production (Saito *et al*. 2010; Yang *et al*. 2017). Among the cytokines considered in the present study, IL‐2, TNF‐α and IFN‐γ are produced by T helper 1 cells and play an important role in the cell‐mediated immune response. By contrast, IL‐4, IL‐6 and IL‐10 are secreted by T helper 2 cells and enhance humoral immunity (Kikuchi and Crystal 2001). Moreover, we have studied the IL‐17 secreted by T helper 17 cells which is involved in allergic responses by inducing and mediating the proinflammatory responses (Korn *et al*. 2009). Overproduction or inappropriate production of certain cytokines by the body can result in inflammatory diseases.

Occasionally, it has been reported that the insertion of external bacterial cells inside the human body leads to inappropriate production of certain cytokines which can cause inflammatory diseases (Percoco *et al*. 2013; Cattaneo *et al*. 2017). In our study, the group of mice treated with *L. paracasei* DTA81 showed lower average values for IL‐6 and IL‐10 when compared to CD and HFD groups. Cytokine IL‐6 is locally produced in response to infection or injury and delivered to other body parts by the bloodstream, activating immunological defences. IL‐6 also stimulates intestinal epithelial proliferation and repair after injury (Kuhn *et al*. 2014); however, excessive or prolonged production of IL‐6 is involved in various diseases (Narazaki and Kishimoto 2018). Several studies have reported that IL‐6 is a pro‐inflammatory cytokine that is detected in higher amounts in obese individuals and contributes to the occurrence of type 2 diabetes, insulin resistance and CVDs (Higa and Panee 2011; Shi *et al*. 2019). The IL‐6 level observed in this work does not differ between CD and HFD groups and can be explained by the reduced duration of the experiment when compared to other studies in the literature assessing the immunomodulatory effect of probiotics (8–14 weeks) (Sheil *et al*. 2006; Antunes *et al*. 2020). Moreover, it can be related to a potential pro‐inflammatory property of the commercial diet, which has approximately 56% carbohydrate, a component that at high concentrations induces inflammation in skeletal muscle (Antunes *et al*. 2020). As regards the reduction of IL‐10, it could be explained considering the strong positive correlation between IL‐6 and IL‐10 normally found in the human body, which contributes to homeostasis maintenance (Dizdarević‐Hudić *et al*. 2009; Sapan *et al*. 2016). The IL‐10 level was reduced by DTA81 intake, and a significant difference was observed compared to the other two groups. Although the increase in IL‐10 level is referred to as the immunomodulatory effect of probiotics, this is not the unique form of probiotics action, which depends on the bacterial strain, concentration and administration method. The reduction level of IL‐10 may decrease the immunostimulatory effect of this cytokine in innate immunity (de Moreno de LeBlanc *et al*. 2011).

It is well known that probiotic traits are strain‐specific and this gives strong motivation to keep seeking new potentially better strains (Senok *et al*. 2005). In our study, L. paracasei DTA81 indicated a good resistance to the gastrointestinal environment in‐vivo. The resistance of lactobacilli to the harsh GIT conditions reported previously (Noriega *et al*. 2004; Burns *et al*. 2010) seems to be linked to the preservation of cell internal pH, functionality and integrity of cell membrane, and to the existence of bile salt efflux pumps (Bustos *et al*. 2011; Wu *et al*. 2012, 2014).

The outcomes of this study revealed the possible mechanism that started from the gut microbiota regulation by *L. paracasei* DTA81 with increasing the abundance of *Bacteriodetes* and *Coprococcus* taxa followed by a significant reduction of inflammatory cytokine interleukin 6 which has subsequently led to a decrement of FBS. On the other side, total cholesterol reduction in the group treated with DTA81 could be possibly related to the above‐mentioned gut microbiota modulation as well as the direct cholesterol assimilation by *L. paracasei* DTA81. Overall, considering the results of this study alongside the previous findings, *L. paracasei* DTA81 has a great potential to be used as a commercial promising probiotic with great influence on metabolic homeostasis in individuals, particularly those adopting high‐fat diets.

## Conflict of Interest

No conflict of interest declared.

## Author Contributions

Conceptualization: A.T., B.C.S.C. and V.S.D.; investigation: A.T., B.C.S.C., V.S.D., R.S.D., S.P., L.L.O.; data curation: A.T. and V.S.D.; writing—original draft preparation: A.T.; writing—review and editing: A.T. and A.G.; supervision: A.G., S.O.P., M.C.G.P., L.L.O.; funding acquisition: A.G., V.C., S. O. P. and M.C.G.P.; All authors have read and agreed to the published version of the manuscript.

## Supporting information

Figure S1. Rarefaction curves of Shannon entropy (a) and phylogenetic diversity (b) of stool samples before (t0) and after (t1) 6 weeks of experimental period.
Figure S2. Relative abundance of bacterial phyla (a), top‐20 families (b), and top‐20 genera (c) identified in feces samples of BALB/c mice in groups CD, DTA81 and HFD before (t0) and after (t1) the experimental period.
