# Supplementary material for: Lactobacillus paracasei DTA81, a cholesterol‐lowering strain having immunomodulatory activity, reveals gut microbiota regulation capability in BALB/c mice receiving high‐fat diet
Source: J Appl Microbiol. 2021 May 6;131(4):1942–57. doi: 10.1111/jam.15058 (PMC8518695; doi:10.1111/jam.15058)

***Lactobacillus paracasei* DTA81,** **a cholesterol-lowering strain with immunomodulatory activity, revealed gut microbiota regulation in BALB/c mice receiving a high-fat diet**

Armin Tarrah^1^, Bruna Cristina dos Santos Cruz^2^**,** Roberto Sousa Dias^3^, Vinícius da Silva Duarte^1^, Shadi Pakroo^1^, Sérgio Oliveira de Paula^3^**,** Leandro Licursi de Oliveira^3^**,** Maria do Carmo Gouveia Peluzio^2^, Viviana Corich^1^, Alessio Giacomini^1^

^1^ Department of Agronomy Food Natural Resources Animals and Environment, University of Padova, Viale dell’Universitá, 16, 35020 Legnaro (PD), Italy

^2^ Department of Nutrition and Health, Federal University of Viçosa, Av. Peter Henry Rolfs, s/n, Campus Universitário, 36570-900, Viçosa, Minas Gerais, Brazil

^3^ Department of General Biology, Federal University of Viçosa, Av. Peter Henry Rolfs, s/n, Campus Universitário, 36570-900, Viçosa, Minas Gerais, Brazil

**Supplementary Figure 1** – Rarefaction curves of Shannon entropy (A) and Phylogenetic diversity (B) of stool samples before (t0) and after (t1) six weeks of experimental period. CD – conventional diet group; DTA81 – *L. paracasei* DTA81 group; HFD – High-fat diet group.


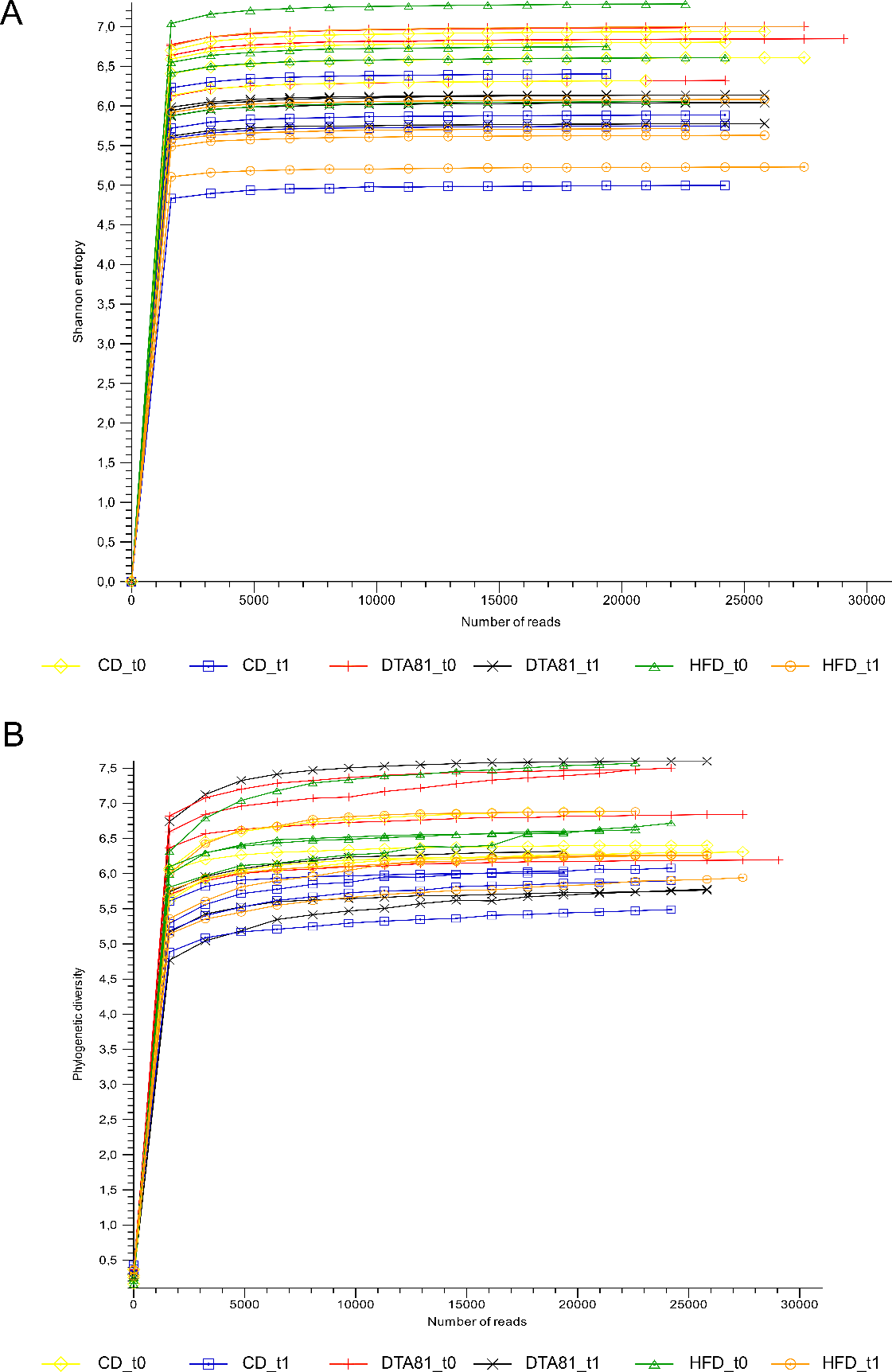


**Supplementary Figure 2** - Relative abundance of bacterial phyla (A), top-20 families (B), and top-20 genera (C) identified in feces samples of BALB/c mice in groups CD, DTA81 and HFD before (t0) and after (t1) the experimental period. Taxa were sorted by the decreasing order of their average relative abundance.


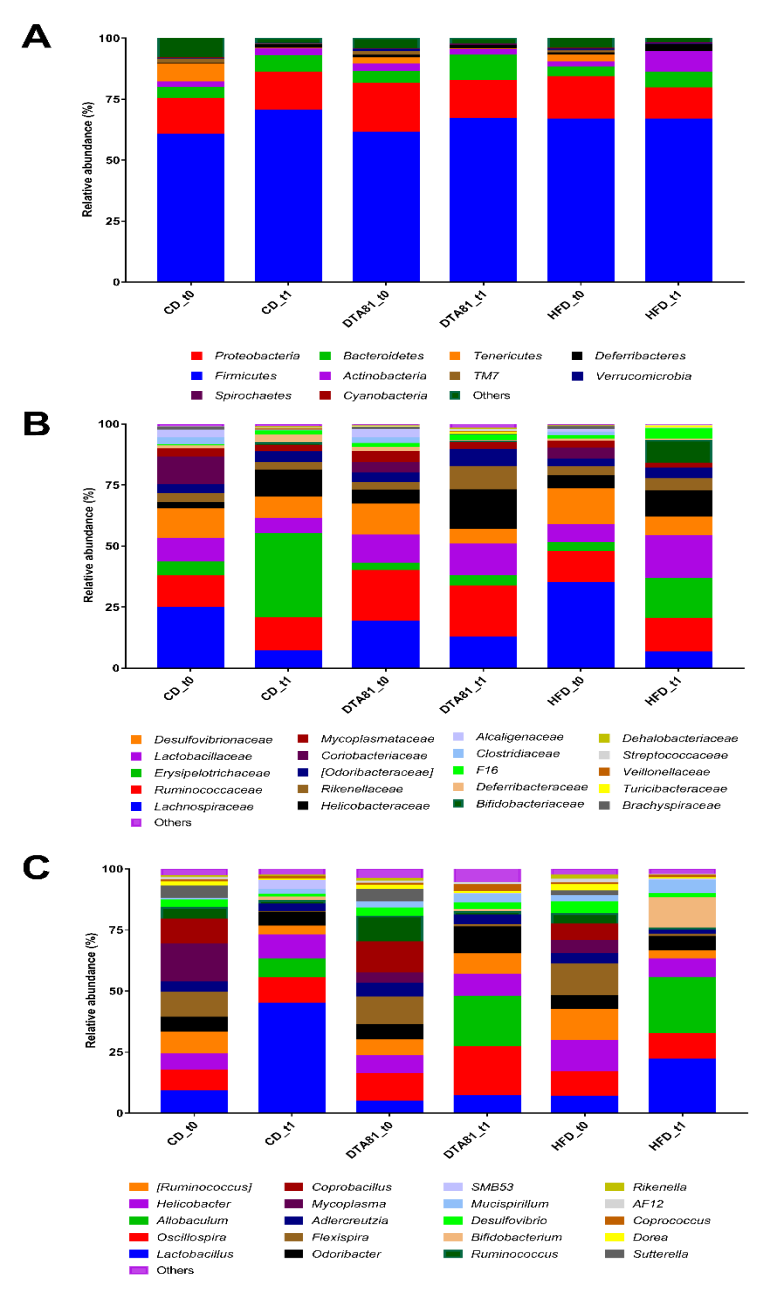

Supplement: Supplementary file 1 — Figure S1. Rarefaction curves of Shannon entropy (a) and phylogenetic diversity (b) of stool samples before (t0) and after (t1) 6 weeks of experimental period. Figure S2. Relative abundance of bacterial phyla (a), top‐20 families (b), and top‐20 genera (c) identified in feces samples of BALB/c mice in groups CD, DTA81 and HFD before (t0) and after (t1) the experimental period. [file JAM-131-1942-s001.docx]
